# Biosynthesis of exopolysaccharide from waste molasses using *Pantoea* sp. BCCS 001 GH: a kinetic and optimization study

**DOI:** 10.1038/s41598-022-14417-1

**Published:** 2022-06-16

**Authors:** Seyyed Vahid Niknezhad, Sedigheh Kianpour, Sina Jafarzadeh, Mohsen Alishahi, Ghasem Najafpour Darzi, Mohammad Hossein Morowvat, Younes Ghasemi, Amin Shavandi

**Affiliations:** 1 Burn and Wound Healing Research Center, Shiraz University of Medical Sciences, Shiraz, 71987‐54361 Iran; 2 Pharmaceutical Sciences Research Center, Shiraz University of Medical Sciences, P.O. Box 71468-64685, Shiraz, Iran; 3 Department of Energy Conversion and Storage, Technical University of Denmark, Fysikvej, 2800 Kgs, Lyngby, Denmark; 4 Department of Chemical Engineering, Faculty of Engineering, Noshirvani University of Technology, Babol, Iran; 5 Department of Pharmaceutical Biotechnology, School of Pharmacy, Shiraz University of Medical Sciences, P.O. Box 71468-64685, Shiraz, Iran; 6 Université Libre de Bruxelles (ULB), École Polytechnique de Bruxelles-BioMatter Unit, Avenue F. D. Roosevelt, 50-CP 165/61, 1050 Brussels, Belgium

**Keywords:** Biological techniques, Biotechnology, Engineering

## Abstract

The bacterium *Pantoea* sp. BCCS 001 GH produces an exopolysaccharide (EPS) named Pantoan through using sugar beet molasses (SBM) as an inexpensive and widely available carbon source. This study aims to investigate the kinetics and optimization of the Pantoan biosynthesis using *Pantoea* sp. BCCS 001 GH in submerged culture. During kinetics studies, the logistic model and Luedeking–Piret equation are precisely fit with the obtained experimental data. The response surface methodology (RSM)-central composite design (CCD) method is applied to evaluate the effects of four factors (SBM, peptone, Na_2_HPO_4_, and Triton X-100) on the concentration of Pantoan in batch culture of *Pantoea* sp. BCCS 001 GH. The experimental and predicted maximum Pantoan production yields are found 9.9 ± 0.5 and 10.30 g/L, respectively, and the best prediction factor concentrations are achieved at 31.5 g/L SBM, 2.73 g/L peptone, 3 g/L Na_2_HPO_4,_ and 0.32 g/L Triton X-100 after 48 h of submerged culture fermentation, at 30 °C. The functional groups and major monosaccharides (glucose and galactose) of a purified Pantoan are described and confirmed by ^1^HNMR and FTIR. The produced Pantoan is also characterized by thermogravimetric analysis and the rheological properties of the biopolymer are investigated. The present work guides the design and optimization of the *Pantoea* sp. BCCS 001 GH culture media, to be fine-tuned and applied to invaluable EPS, which can be applicable in food and biotechnology applications.

## Introduction

Bacterial exopolysaccharides (EPSs), biologically synthesized by various bacteria, are polymeric carbohydrate molecules with a long chain of monosaccharides. High production capacity, low resource-intensiveness, and the physicochemical and structural properties of bacterial EPSs make them interesting for a wide range of industrial applications, for instance in textile, cosmetics, medicine, pharmaceuticals, petrochemical, and food^1–9^. However, due to the high cost of production, only a few bacterial EPSs such as xanthan, dextran, gellan, and curdlan have entered the market^10^. Therefore, identifying new bacteria species with high EPS yield is necessary. Low-cost substrates such as cheese whey, molasses, starch, saline by-products, and glycerol usually include high amounts of carbohydrates that have been previously utilized to produce EPSs by bacteria^11–16^. *Pantoea*, a gram-negative bacteria from the *Enterobacteriaceae* family has several species such as *Pantoea agglomerans*, *Pantoea* sp*., Pantoea alhagi*, and *Pantoea stewartii* that can produce EPSs with diverse compositions and carbohydrate structures and hold an immense promise as a new microorganism for industrial applications^17–23^.

Microbial EPSs such as pullulan, levan, xanthan gum have gelling, emulsifying and prebiotic properties with antioxidant, antimicrobial, anticancer, and blood cholesterol-lowering activities, which make them beneficial to use as food additives^24–27^. Therefore, microbial EPS have been widely used in the food and pharmaceutical and medical industries^28–31^. These polymers can be utilized as gelling, emulsifier, texturizers and stabilizers agents for dairy products^32–34^, bakery products^35–37^ and yogurt-like beverages and cheese alternatives^38,39^. Also, as fermentation agents and alternatives to the hydrocolloids and phosphates can be used to improve the quality of the meat products^40,41^. Considering their chemical and physical structures, these polymers can be used for thickening, suspending, and gelling applications^28^. Besides prebiotic properties, these polymers possess suitable features such as antioxidant, antimicrobial, anticancer, and blood cholesterol-lowering activities, which make them beneficial to use as food additives^24^. Mathematical modeling of the fermentation process is a powerful tool for better understanding the EPSs’ fermentation procedure and determining the significance of its variables^14,42^. Response surface methodology (RSM) is an efficient mathematical and statistical technique, which is aimed at optimizing the condition of factors for required responses and studying the relative significance of the affecting factors. This method can be carried out even in the presence of compound interactions, which have a significant impact on the yield of the final product^42,43^. A growth kinetic model is another method that can be particularly useful to describe the behavior of the cellular procedures through substrate utilization and fermentation product. Numerous models also throughout possible mathematical equations have been recommended to be an extremely efficient instrument to test and eliminate the extremities^44,45^. RSM and unstructured growth kinetic models have been used for modeling, optimizing the bioprocesses, and expanding the stoichiometric relationship between variables namely cell growth, substrate utilization, and product formation. These methods have been extensively applied in numerous biotechnology products, for instance, xanthan gum, EPS production by *Enterobacter* A47 and *Pseudomonas fluorescens* CrN6, and ethanol production^42,44,46,47^.

In this study, Pantoan was produced by using *Pantoea* sp. BCCS 001 GH^2,17^. The focus of present work is on kinetics, optimization, determination of the interaction among fermentation parameters, and characterization of Pantoan biosynthesis using *Pantoea* sp. BCCS 001 GH in submerged culture. Additionally, the medium variables such as molasses concentration (without supplementary carbon source added), nitrogen source, phosphor, and Triton X-100 have been optimized to maximize the yield of Pantoan using RSM. The kinetics of the process has been thoroughly investigated in the presence of various concentrations of molasses—a cost-effective and alternative substrate–and the stoichiometric relationship between the variables namely cell growth, substrate consumption, and Pantoan production was determined. To further classify Pantoan, the monomer composition, physicochemical and rheological properties was analyzed.

## Materials and methods

### Beet molasses characterization

Molasses as a by-product of the sugar beet industry was provided by the Marvdasht sugar factory (Marvdasht, Iran). The concentration of sugars in the diluted beet molasses was determined using high-pressure liquid chromatography (HPLC, Waters 600, 2414, USA) with a Knauer Eurokat column and RI detector. In brief, 20 μL of diluted beet molasses was injected into the column and the mobile phase composed of 0.01 N H_2_SO_4_ with a flow rate of 1 mL/min was used for elution at room temperature. The standard solutions of different sugars were prepared by dissolving different amounts of standard sugars in water and analyzed with HPLC. The amounts of fructose and glucose were estimated in the initial beet molasses. The diluted beet molasses was autoclaved at 121 °C for 20 min before fermentation. Dinitrosalicylic acid (DNS) assay was employed to measure liberated SBM before and during the fermentation process. The total consumed sugar during fermentation was determined as maltose equivalents by a colorimetric method^48^. Finally, the diluted molasses was centrifuged at 10,000 rpm for 10 min, and the concentration of Ca^2+^, Cu^2+^, Zn^2+^, Fe^2+^, and Pb^2+^ were determined using an atomic absorption spectrophotometer (AAS) (SpectrAA 220, Varian, USA). The standard solutions were prepared by dissolving different amounts of standard ions in Milli-Q water and analyzed with AAS.

### Microorganisms and inoculums preparation

*Pantoea* sp. BCCS 001 GH was acquired from the culture collection of the Pharmaceutical Sciences Research Center, Shiraz University of Medical Sciences (Shiraz, Iran). The bacteria was cultivated on YPD (yeast extract (1% w/v), peptone (2% w/v), and glucose (2% w/v)) at pH 6.5 and 30 °C for 48 h later used as an inoculum for producing Pantoan^2^. The inoculums (2% v/v) were added to the cotton plugged Erlenmeyer flasks containing 50 mL of an autoclaved solution. A set of culture solutions was used to check the effect of molasses concentration on the kinetic and yield of Pantoan. A second set of culture solutions were used for the optimization studies. The first culture solution contained 3 g/L of peptone, 3 g/L of Na_2_HPO_4_, 0.006 g/L of H_3_BO_3_, 0.006 g/L of ZnCl_2_, 0.002 g/L of FeCl_3_, and 0.2 g/L of Triton X-100, the pH of the solutions was adjusted to 6.5 and autoclaved at 121 °C for 20 min. Flasks were supplemented with SBM (total sugars: 10–40 g/L). For the second culture solutions which contained 0.006 g/L of H_3_BO_3_, 0.006 g/L of ZnCl_2_, and 0.002 g/L of FeCl_3_ were adjusted to pH 6.5 and autoclaved at 121 °C for 20 min. Flasks were supplemented with SBM, and the amounts of peptone, Na_2_HPO_4_, and Triton X-100 were adjusted according to Table 1. The pH values of the solutions were adjusted to 6.5, by adding 1 N NaOH before the autoclaving. The SBM was also autoclaved separately and added to the culture medium. The cultivation was performed at 30 °C and 200 rpm for 48 h. The Pantoan extraction and purification was performed following our previous reports^2,17^.

**Table 1:** Experimental range and variable levels for second culture.

Name/factor	Molasses g/L (A)	Peptone (g/L (B)	Na_2_HPO_4_ g/L (C)	Triton X-100 mg/L (D)
Low level	10 (− 1)	1 (− 1)	1 (− 1)	50 (− 1)
	25	3	3.5	225
High level	40 (+ 1)	5 (+ 1)	6 (+ 1)	400 (+ 1)

### Kinetics analysis and modeling

The kinetics mathematical model is a powerful tool to anticipate the fermentation reactions involved in scaling up a product. In this study, fundamental unstructured kinetic models were used for cell growth, product synthesis, and substrates consumption. In this research, the rate equation is stated by the process variables—cell mass concentration (x), product concentration (p), and sugar concentration of substrate (s) during Pantoan fermentation.

### Growth dynamics

The unstructured kinetic model (logistic kinetic equation) was used for microbial growth, which is a viable kinetic model to forecast the growth rate by using a contributed theory of Verhulst in 1844, and Pearl and Reed in 1920, which include an inhibiting factor to population growth. To assume that inhibition is proportional to x_s_, Eq. (1) is used, stated as follows:

1 $$\frac{dx}{dt}=kx\left(1\frac{x}{{x}_{s}}\right)$$

where t is the development time (h), x is the cell mass concentration (g/L), x_s_ is the saturated cell mass, k is the carrying capacity (cell mass the fermentation broth can hold). The logistic curve is sigmoidal and leads to a stationary population of size, x_s_ = 1/β. The above equation is used to predict cell growth in batch experiments. By using simple algebraic manipulations, Eq. (2) can be written as:

2 $$x=\left(\frac{{x}_{0}{x}_{max}{e}^{kt}}{{{x}_{max}-x}_{0}{+x}_{0 }\;{e}^{kt}}\right)$$

### Product formation kinetics

A typical and widely used product kinetic model is Luedeking–Piret model, an unstructured method composed of two terms which are considered as growth-associated and non-growth-associated constants for product formation^45^. According to this model, the product formation rate depends linearly on the growth rate, and the cell concentration

3 $$\frac{dp}{dt}=\alpha \frac{dx}{dt}+ \beta x$$

where α and β are product formation constants contributing to growth associated and non-growth associated fermentation conditions and depend on the fermentation dynamics. The product formation rate, dp/dt, reveals the correlation between cell mass and product concentration. Consequently, by the use of simple algebraic manipulations for product formation and incorporating Eq. (2) into (3); the resulting Eq. (4) can be written as follows with initial condition t = 0, P = P_0_:

4 $$p={p}_{0}+\alpha {x}_{0}\left(\frac{{e}^{kt}}{1-\frac{{x}_{0}}{{x}_{max}}(1-{e}^{kt})}-1\right)+\beta \frac{{x}_{max}}{k}\mathrm{ln}\left[1-\frac{{x}_{0}}{{x}_{max}}\left(1-{e}^{kt}\right)\right]$$

### Substrate consumption kinetics

Substrate utilization kinetics is given as the modification of the Luedeking–Piret model, considering substrate conversion to cell mass, to product and substrate consumption as stated in Eq. (5)

5 $$\frac{ds}{dt}=-\frac{1}{{Y}_\frac{x}{s}}\frac{dx}{dt}-\frac{1}{{Y}_\frac{p}{s}}\frac{dp}{dt}+ {k}_{e}x$$

Y_x/s_ is the yield coefficient for biomass with respect to substrate consumption, and Y_p/s_ is the yield coefficient for formed products with respect to substrate consumption. To simplify the equation, two constant terms are defined as:

6 $$\gamma=-\frac{1}{{Y}_\frac{x}{s}}+\frac{\alpha }{{Y}_\frac{p}{s}} \; and \; \eta=\frac{\beta }{{Y}_\frac{p}{s}} + {k}_{e}$$

Substituting Eq. (6) into Eq. (5) and integration, the following differential equation is obtained for the substrate concentration:

7 $$\frac{ds}{dt}=-\gamma \frac{dx}{dt}-{\eta} x$$

where γ and η are considered as growth and non-growth associated constants, respectively^49^. When integrating the above equation using initial condition S = S_0_ at t = 0, and implementing Eq. (2) into Eq. (7), the substrate utilization equation will be obtained as follows:

8 $$S={S}_{0}-{\gamma}{x}_{0}\left(\frac{{e}^{kt}}{1-\left(\frac{{x}_{0}}{{x}_{max}}\right)\left(1-{e}^{kt}\right)}-1\right)- \eta \frac{{x}_{m}}{k}\mathrm{ln}\left(1-\frac{{x}_{0}}{{x}_{max}}\left(1-{e}^{kt}\right)\right)$$

The experiment data analysis and drawing of the graphs were performed by using Origin Pro (2017 SR2 Build 380, Origin Lab Corporation, USA) software. Therefore, non-linear curves of the Eqs. (2, 4, and 8) were fitted to the experimental data, and the Curve Fitting Toolbox of the software was used to study the performance of cell growth concentration, Pantoan production, and SBM substrate consumption.

### Central composite design (CCD)

Carbon source, peptone, Na_2_HPO_4,_ and Triton X-100 are the major process parameters affecting on the yield of Pantoan in culture media 19. In this study, a central composite design was utilized to optimize the four important variables that considerably affected Pantoan production. Design Expert software (Version 10.0.7.0, Stat-Ease Inc., Minneapolis, USA) was used to frame the experimental designs and statistical analysis. Exclusively, the influences of four independent variables were evaluated at three levels (− 1, 0, + 1) with 21 experimental runs and 5 repetitive central points (small type CCD; Table 1). Then, the experiments were conducted in 250 mL Erlenmeyer flasks with 50 mL of media, under 200 rpm agitating at 30 °C for 48 h, prepared according to the design. Later, the response obtained from RSM could be represented by a second-order polynomial equation as follows:

9 $$Y = \beta_{0} + \beta_{1} A + \beta_{2} B \, + \beta_{3} C \, + \, \beta_{4} D \, + \, \beta_{12} AB \, + \beta_{13} AC \, + \beta_{14} AD \, + \beta_{23} BC + \beta_{24} BD \, + \beta_{34} CD$$

where Y is the predicted response (Pantoan concentration), A, B, C, and D represent the levels of the factors according to Table 1, and β_0_, β_1_,…, β_34_ represent coefficients estimates with β_0_ having the role of an intercept constant. Then, the experiments were carried out in triplicates. Since the response (yield of Pantoan g/L) was the dependent variable, the obtained 2D graphical plots would illustrate the mutual interactions between determinative factors, thus, evaluating the optimal medium components.

### Physicochemical characterization of Pantoan

The presence of functional groups in the isolated Pantoan sample was preliminary confirmed using a Fourier transform infrared spectrophotometer (Vertex 70/70v FT-IR, Bruker, USA). A pressed pellet of 1 mg Pantoan was prepared with 100 mg potassium bromide (KBr) and scanned in the transmittance (%) mode with a resolution of 4/cm range from 400 to 4000/cm. Then, Ultraviolet–Visible (UV–Vis) spectroscopy analysis of the Pantoan was conducted using a UV–Vis spectrophotometer (Cary Series UV/Vis Spectrometer, Agilent Technologies, USA) in the wavelength range of 200–700 nm, to confirm the presence of nucleic acids and proteins in the Pantoan EPS samples^17^. The X-ray diffraction (XRD) study was accomplished based on the previous studies in the 2Ø range of 5–40 with seed size of 0.05 and step time of 1 s on the Bruker D8 Advance model XRD with Ni-filtered Cu Kα radiation generated at 40 kV and 40 mA^17^. ^1^H NMR spectrum of Pantoan was acquired using an Ascend™ Bruker 400 MHz spectrometer (Bruker Corporation, Switzerland) at 25 °C to investigate the monomers of the polysaccharide. Afterward, the sample was dissolved in D_2_O at the concentration of 20 mg/mL and the proton pulse was adjusted to 14.35 μs while the time acquisition was 4.08 s. Chemical shifts (δ) were described in parts per million (ppm). The thermal stability of the biosynthetic Pantoan from beet molasses was determined by Thermogravimetric analysis (TGA) using a TA TGA Q500 Thermogravimetric analyzer ^50^. The experiment was conducted at the heating rate of 10 °C/min, under a nitrogen atmosphere to determine the mass loss; in the temperature range of 25–900 °C. Lastly, the dynamic variance scanning calorimetry (DSC) experiment using TA DSC Q200 differential scanning calorimeter (DSC, USA) was carried out in the temperature range of − 50 to 400 °C at a ramping up rate of 10 °C/min and under nitrogen flow, in order to investigate the response of the polymer to heating.

### Rheological study

The rheological properties of Pantoan were analyzed on an HR-2 Discovery Hybrid rheometer (TA Instruments, USA) equipped with parallel plate geometry (20 mm diameter, 0.5 mm gap) at a Peltier-controlled temperature. Biopolymer solutions with different concentrations (1, 1.5, and 2% w/v) were prepared by dissolving Pantoan in Milli-Q water under gentle stirring at room temperature. To investigate the flow behavior of the sample, steady shear viscosity was measured under the shear rate between 0.1 and 500/s at 25 °C. For the sake of comparison, xanthan gum as regular exopolysaccharide in the food industry was also examined with the concentration of 1 and 2% w/v. The linear viscoelastic region (LVR) was then determined at 25 °C, implementing an amplitude sweep test from 0.1 to 100% oscillation strain at a constant angular frequency of 10 rad/s. Additionally, the rheological behavior of the material was examined as a function of temperature between 25 and 40 °C.

### Statistical analysis

The statistical analysis of all data was carried out with GraphPad Prism 9 (GraphPad Software, San Diego, CA) using ordinary one-way ANOVA. The results were reported as the mean value ± SD and comparisons were performed by Tukey's multiple comparisons test (*p* < 0.05).

## Results and discussions

### Fermentation production and kinetic analysis

**Figure 1:**
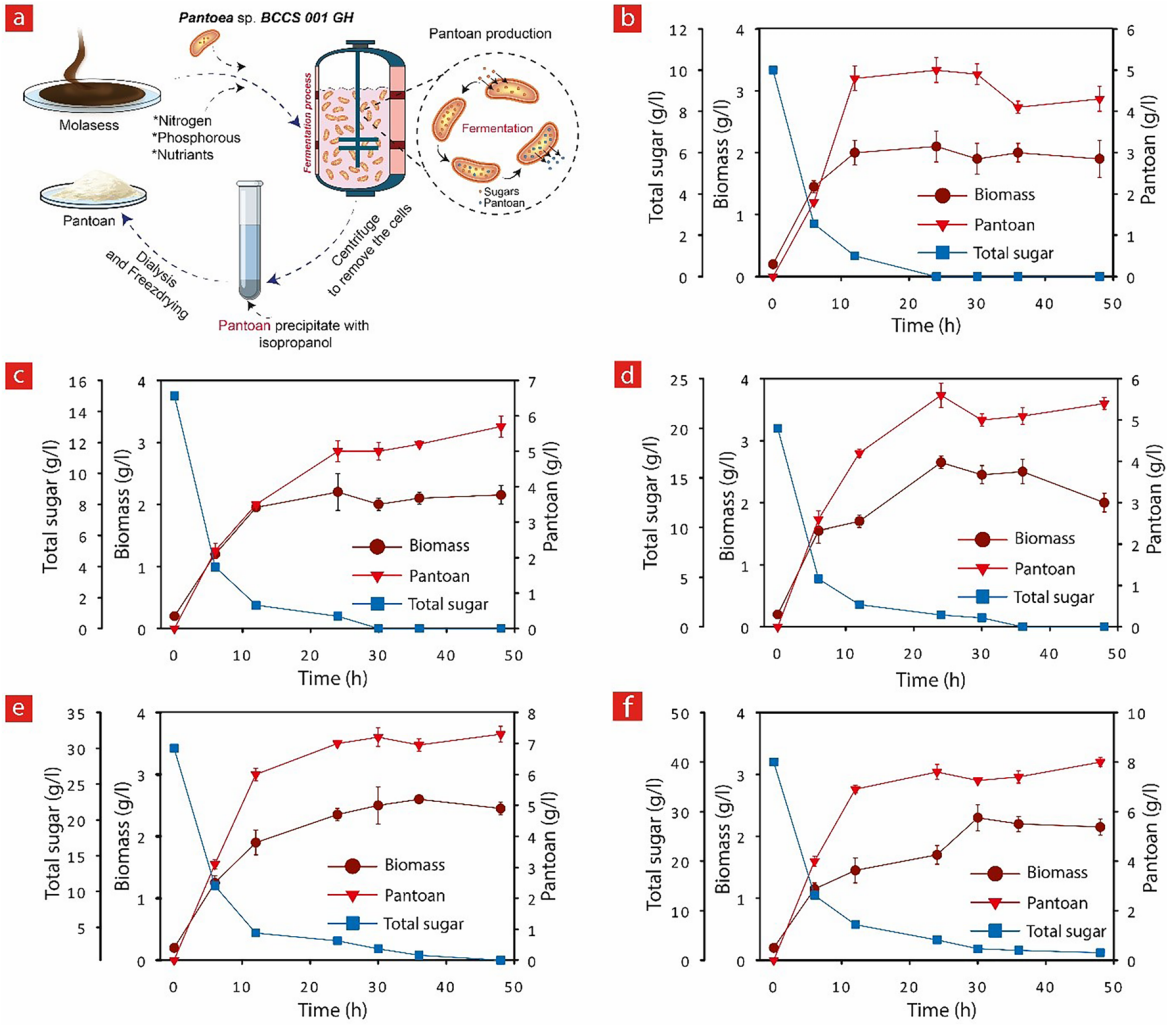
The effect of SBM concentration on biomass and Pantoan production using GraphPad Prism 9 (GraphPad Software, San Diego, CA). (**a**) A schematic showing the production and purification process of Pantoan. (**b**–**f**) Study of biomass and Pantoan production and SMB total sugre consumption (substrate consumption) at 1%, 1.5%, 2%, 3% and 4% SBM.

The amounts of fructose and glucose as major SBM in the beet molasses were found (Table 2) to be 35.14 and 64.85 W%, respectively. To understand the effect of SBM concentration on Pantoan production, SBM at different concentrations (1–4%) was added into the batch culture as a carbon source (Fig. 1).

**Table 2:** Characteristics of sugar beet molasses.

Parameter value	Value
Density (g/L)	1210
Total sugar, g/kg molasses	437.4
Total sugar (g/L)	529.2
Glucose (g/L)	343.3
Fructose (g/L)	186
pH	6.6
Ca	774 ppm
Cu	0.16 ppm
Fe	31.5 ppm
Zn	3.54 ppm
Pb	0.16 ppm

The yield of Pantoan reached 7.3 ± 0.3 and 8.0 ± 0.2 g/L at the end of fermentation with 3 and 4% SBM, which are about 54.7 and 60%, respectively higher than 1% SBM. This can be attributed to the superior influence of the carbon source in most biosynthesis of metabolites, which exclusively affects bacterial growth and EPSs production in the fermentation process^51^. According to Fig. 1, a higher SBM concentration (4%) had a beneficial effect on both the bacteria cell growth (2.3 ± 0.2 g/L) and Pantoan production (8.0 ± 0.2 g/L).

**Figure 2:**
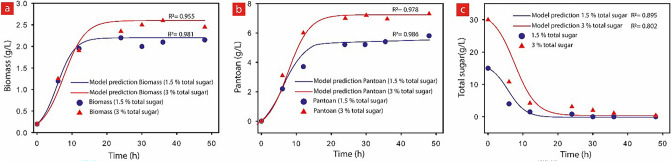
Kinetic studies using GraphPad Prism 9 (GraphPad Software, San Diego, CA).. Experimental (symbols) and modeling results (continuous lines) showing kinetics through time for (**a**) The Biomass, (**b**) Pantoan, and (**c**) total sugar, in the presence of 1.5 and 3% SBM.

In addition, the kinetics of cell growth, substrate consumption, and Pantoan production was modeled and simulated to compare with the experimental data. The classical logistic equation was used to define the cellular growth kinetic and Luedeking–Piret model^52^ was employed to simulate substrate consumption and product formation. The simulations were carried out using Origin Pro (2017, Origin lab Corporation, USA) software. The results obtained from the model prediction and real experiments are compared and presented in Fig. 2 for SBM concentrations of 1.5 and 3%. For the logistic models, the kinetic parameters (k) were calculated using a built-in curve fitting tool kit in the Origin software for each experiment. These results demonstrate acceptable regression and kinetic parameter values for all experiments (k = 0.5, 0.4, 0.32, 0.33 and 0.26 for total SBM concentrations of 1, 1.5, 2, 3 and 4%, respectively). The high association coefficient (R^2^) for all SBM concentrations indicates that the model structure is in conformity with experimental data and the model is appropriate for describing the growths of *Pantoea* sp. BCCS 001 GH^53^. Furthermore, using the obtained ‘k’ values for each experiment (different SBM in total), the values for the stoichiometric coefficients of growth-associated constant and non-growth-associated constant were calculated taking P_0_ as 0, and the results are presented in Table 3. Additionally, the product formation under these diverse fermentation conditions denotes a robust growth and product formation, since the estimated value for the stoichiometric coefficient (α) was found higher than the maintenance coefficient (β). The specific rates for growth and product formation were in proportion with the metabolic activity of the individual cells. Ignoring the lag phase of the fermentation process of Pantoan, the initial growth rate of cells was found to be high (log phase) because cell multiplication was maintained uninterrupted until it reaches the stationary phase due to the depletion of nutrients^44^. Therefore, by neglecting β, the process can be considered as growth associated and all factors such as carbon source, nitrogen source, and nutrient that affect the cell growth would also affect the production of Pantoan^44,54,55^. By employing Luedeking–Piret’s model, the specific relationship between Pantoan production and growth rates was appropriately described with high correlation coefficient (R^2^) values in linear regression plots. In a fermentation process, the study of sugar amount is of great importance, as sugar directly affects the formation and maintenance of the cells as well as the production of the polysaccharides. Thus, in the log phase, the sugar amount instantly reduces while the cell biomass and the amount of Pantoan increases. Equation (8) was used to model the decreasing sugar consumption which is demonstrated in Fig. 1 and the results are presented in Fig. 2 and Table 3. The decreasing sugar consumption seems to be closely growth-associated similar to the product formation, and the growth-associated constant (ϒ) increases 2.83 times when the total SBM concentration varies from 1 to 4%. However, the non-growth-associated constant (η) is about zero suggesting that the decreasing sugar consumption is closely growth-associated when Pantoan fermentation is evaluated by beet molasses as substrate.

**Table 3:** Predicted values of cell mass concentration, substrate utilization, and product formation of *Pantoea* sp. BCCS 001 GH.

Parameters	1%	1.5%	2%	3%	4%
*K*	0.50	0.4	0.32	0.33	0.26
Α	2.36	1.96	2.18	3.02	3.82
Β	~ 0.00	0.02	0	0	0
ϒ	5.24	7.58	8.37	12.35	14.83
Η	0	0	0	0	0.085

### Optimization of Pantoan production and response surface methodology (RSM)

After studying the effect of different parameters, a statistical method was utilized to maximize Pantoan production in the batch flask fermentation through optimizing the procedure variables. To this end, a central composite design with four factors of SBM, peptone, disodium hydrogen phosphate, and Triton X-100 concentrations, were employed^17^. Even though one factor at a time analysis enables evaluating the effect of individual parameters on the Pantoan production, it fails to identify the inter-correlation between two or more effective variables. Therefore, RSM was employed in this study to evaluate the combinatorial influence of the four previously mentioned independent variables. A design model with 21 runs in one block was exhibited and each independent variable was tested at five levels (Table 4). Each run was performed in triplicate and the values of Pantoan yield were compared with the predicted values of responses obtained from the model using the software Design Expert 10.0.7.0.

**Table 4:** A central composite design array with responses (in actual units) for Pantoan production after 48 h of fermentation.

Run	A: Carbon source	B: Nitrogen source	C: Phosphor source	D: Triton X-100 (g/L)	Pantoan (g/L)
	Molasses (g/L)	Peptone (g/L)	Na_2_HPO_4_ (g/L)		
1	25	5.4	3.5	0.225	9.6
2	25	3	3.5	0.435	8.9
3	25	3	0.5	0.225	6
4	10	1	1	0.05	2.2
5	7	3	3.5	0.225	4
6	25	3	3.5	0.225	9
7	10	1	6	0.05	3.6
8	40	5	6	0.05	8.1
9	25	3	3.5	0.225	8.8
10	25	3	3.5	0.225	8.9
11	25	3	3.5	0.015	3.8
12	43	3	3.5	0.225	8.8
13	40	1	1	0.4	7
14	25	3	3.5	0.225	9
15	10	5	1	0.4	4.4
16	40	5	1	0.05	8.24
17	25	0.6	3.5	0.225	6.8
18	25	3	3.5	0.225	8.6
19	25	3	6.5	0.225	8.1
20	40	1	6	0.4	6.8
21	10	5	6	0.4	3.6

Then, a design model was used to define the working region to produce the highest Pantoan yield and determine the optimal conditions for producing Pantoan with a specific composition. Analysis of variance (ANOVA) for the Pantoan is summarized in Table 5. For the whole set of responses (R), the regression analyses showed R^2^ and R^2^_adj_ values of 0.972 and 0.927, respectively, which indicate that the fitted model can explain 97.65 and 92.17% of the variability in Pantoan concentration. The high R^2^ value > 90% suggests that the experimental results (actual data) are in good agreement with theoretical values predicted by the model (Supplementary Fig. 1)^42,56,57^. The ANOVA p-values showed that the second-order model can significantly (*p* < 0.05) predict the Pantoan response. The results show that the selected model can precisely describe the effects of SBM, peptone, disodium hydrogen phosphate, and Triton X-100 concentrations on Pantoan production (R) by *Pantoea* sp. BCCS 001 GH in a batch flask and can be described by the following multiple regression equation:

$$Y=\left[2.91+0.4A+0.2B+0.058C+0.43D+0.41AB-0.35AC+0.070AD-0.075BC-0.047BD-0.078CD\right]$$

where Y is Pantoan production (g/L) and the predicted response variable; and A, B, C, and D are the coded values of the independent variables, namely SBM, peptone, disodium hydrogen phosphate, and Triton X-100 concentrations, respectively. Additionally, variables A, B, D, and AB had significant (95%, *p* < 0.05) positive effects on the Pantoan production, indicating the practicality of these second-order polynomial equations. Nevertheless, regression coefficients for C and AC, AD, BC, BD, and CD were found not significant (*p* > 0.05) and had negative effects on Pantoan yields.

**Table 5:** Analysis of Variance (ANOVA) statistics for Pantoan using sugar beet molasses media.

Source	Pantoan				
	Degrees of freedom	Sum of squares	Mean square	F value	*p* value
Model	14	4.61	0.33	17.83	0.0010*
A-Carbon source	1	0.47	0.47	25.29	0.0024*
B-Nitrogen source	1	0.12	0.12	6.52	0.0433*
C-Phosphor source	1	0.036	0.036	1.96	0.2113
D-Triton X-100	1	0.53	0.53	28.94	0.0017*
AB	1	0.36	0.36	19.45	0.0045*
AC	1	9.552E − 003	9.552E − 003	0.52	0.4991
AD	1	0.010	0.010	0.56	0.4833
BC	1	0.045	0.045	2.44	0.1691
BD	1	4.599E − 003	4.599E − 003	0.25	0.6355
CD	1	0.049	0.049	2.67	0.1535
(R^2^, R^2^_adj_) = (97.65%, 92.17%)					

*Significant model terms.

**Figure 3:**
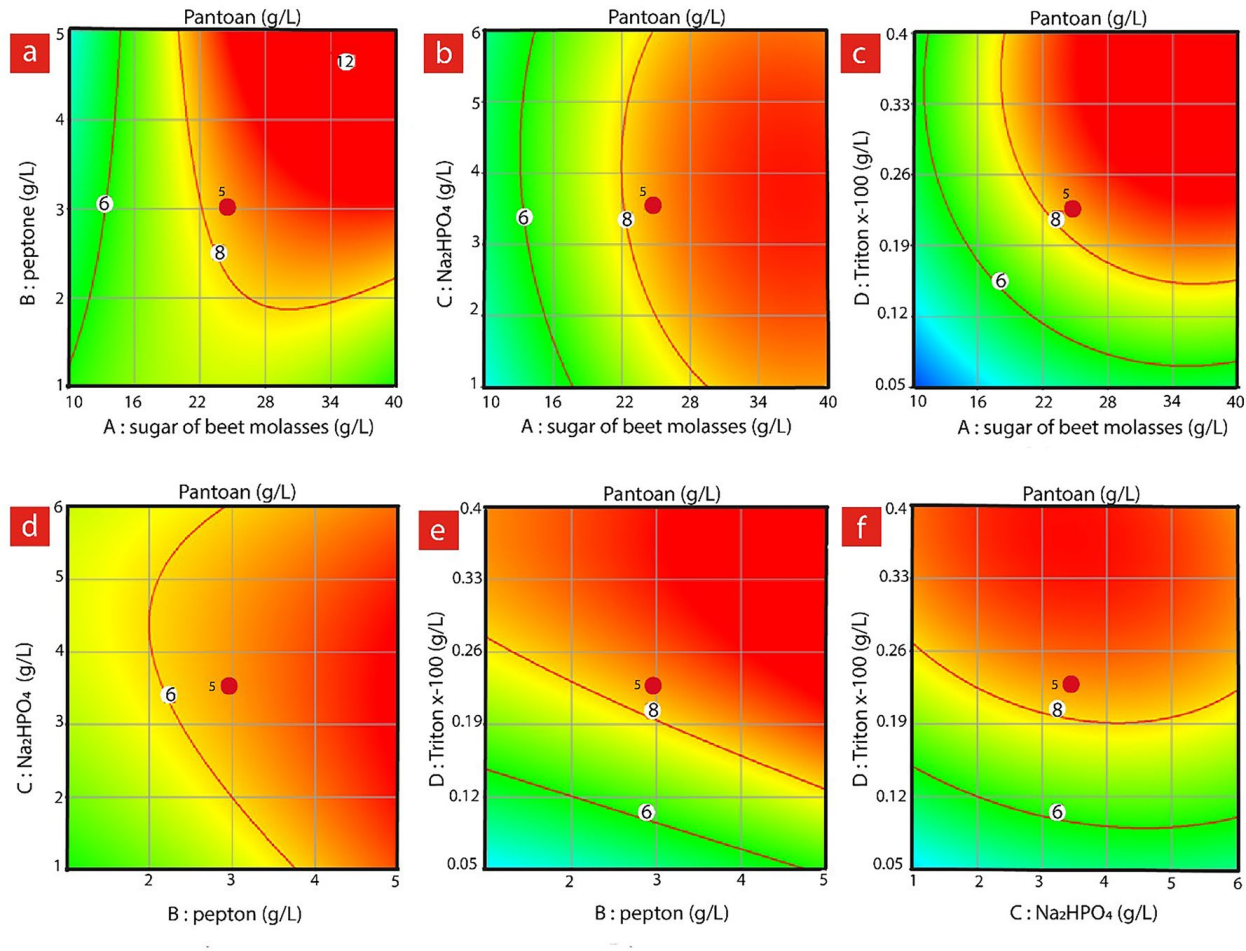
Effect of different concentrations of SBM, peptone, Na_2_HPO_4_, and Triton X-100 on Pantoan production using Design Expert software (Version 10.0.7.0, Stat-Ease Inc., Minneapolis, USA). The rate of production is shown based on the lowest (green color), medium (yellow), and highest (red color). (**a**) concentrations (g/L) of the peptone and SBM at constant concentration of Na_2_HPO_4_, and Triton X-100, (**b**) concentrations of the Na_2_HPO_4_ and SBM at constant concentrations of peptone and Triton X-100, (**c**) concentrations (g/L) of the Triton X-100 and the molasses at constant concentrations of peptone and Na_2_HPO_4_, (**d**) concentrations (g/L) of the peptone and the Na_2_HPO_4_ at constant concentrations of Triton X-100 and molasses, (**e**) concentrations (g/L) of the peptone and the Triton X-100 at constant concentrations of Na_2_HPO_4_ and SBM, and (**f**) concentrations (g/L) of the Na_2_HPO_4_ and the Triton X-100 at constant concentrations of peptone and SBM.

Figure 3 presents the circular contour plots (two-dimensional (2D) plots) of response surfaces of Pantoan concentration for each pair of factors, while the other factors are kept constant at their middle levels. As shown in Fig. 3a, for the various levels of SBM and peptone, the concentration of Pantoan increases from the medium to high SBM and peptone levels, while the concentration of disodium hydrogen phosphate and Triton X-100 was kept constant at medium level. Moreover, Fig. 3b indicates that Pantoan production remains almost constant when increasing the disodium hydrogen phosphate and SBM concentrations at the constant medium level of peptone, and Triton X-100. Pantoan concentration was found to increase with increasing the SBM and Triton X-100 concentrations as demonstrated in Fig. 3c. Additionally, increases in the concentration of all factors (except disodium hydrogen phosphate) result in high Pantoan concentration (Fig. 3d–f). As a final point, the concentration of disodium hydrogen phosphate was found to play no significant role in the production of Pantoan, according to the results demonstrated in Fig. 3b,d (*p*_value_ = 0.211).

**Figure 4:**
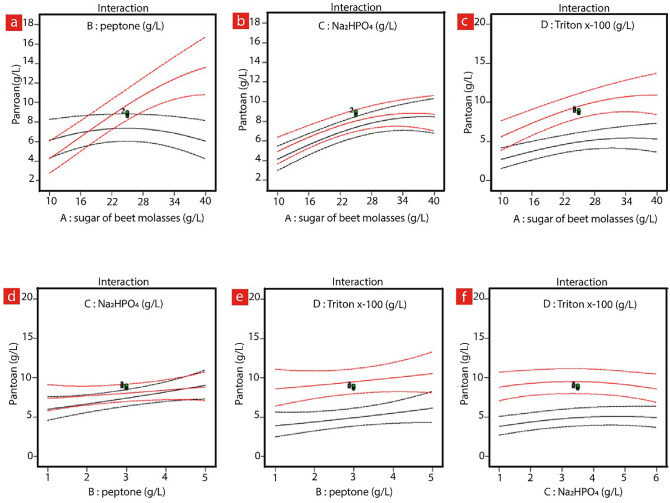
The interaction effects between all factors on Pantoan production using Design Expert software (Version 10.0.7.0, Stat-Ease Inc., Minneapolis, USA). (**a**) Interaction between the peptone and SBM. (**b**) interaction between the Na_2_HPO_4_ and the SBM, (**c**) interaction between the Triton X-100 and SBM, (**d**) interaction between the peptone and the Na_2_HPO_4_. (**e**) Interaction between the peptone and the Triton X-100, and (**f**) interaction between the Na_2_HPO_4_ and the Triton X-100.In order to acquire the maximum Pantoan concentration and therefore the optimum levels of SBM, peptone, disodium hydrogen phosphate, and Triton X-100, a second-order polynomial model was employed to determine the values of these variables. According to the predicted conditions given by the model, the maximum Pantoan yield (10.30 g/L) is obtained at the concentrations of 31.5, 2.73, 2.99, and 0.32 g/L for SBM, peptone, disodium hydrogen phosphate, and Triton X-100, respectively. These numbers illustrate the optimal condition for Pantoan production from molasses by *Pantoea* sp. BCCS 001 GH in a batch flask culture.

The linear plots are shown in Fig. 4, depicting the correlation between two variables while keeping the other variables at zero levels. The comparison between the shapes of contour circular or elliptical plots (Fig. 3), and linear interaction plots (Fig. 4) reveals the significance of the mutual communications between the variables. As shown in Fig. 4, the only significant mutual connection is between SBM and peptone, and since the red and black lines in Fig. 4a are not behaving similarly, the yield of Pantoan is tremendously affected by SBM and peptone concentrations in the range of 10–40 g/L and 1–5 g/L, respectively.

The predicted optimum conditions were applied for the justification experiment, and the actual (experimental) yield value was slightly lower than the predicted value after 48 h of fermentation (~ 9.9 ± 0.5 g/L). To corroborate these results, a confirmation experiment was carried out under the optimum conditions, and finally, it was associated with the kinetic experiments. The design matrix of the independent variables along with the experimental result was reported for Pantoan yield (Supplementary Table 1). This optimization strategy led to an enhanced Pantoan production from non-optimized medium conditions with 30 and 40 g/L SBM, with the yield of 7.3 ± 0.25 and 8.0 ± 0.2 g/L, respectively; to an optimized medium condition, with the yield of 9.9 ± 0.5 g/L. Based on these findings, it can be concluded that the Pantoan yield from *Pantoea* sp. BCCS 001 GH is higher than other microorganisms such as *Paenibacillus polymyxa* SQR-21(3.42 g/L)^58^, *Zunongwangia profunda* SM-A87 (8.9 g/L)^43^, *Komagataeibacter xylinus* BPR 2001 (7.5 g/L)^55^, and *Pseudomonas fluorescens* CrN6 (4.62 g/L)^44^. These results indicate that SBM can serve as an alternative carbon source in the fermentation process to produce Pantoan from *Pantoea* sp. BCCS 001 GH with a higher yield.

### Characterizations of Pantoan

To recognize the structure, functional groups, and surface compounds of the biosynthesized Pantoan, the FTIR spectrum was analyzed as shown in Supplementary Fig. 2a. The EPS possesses a significant number of hydroxyl groups, displayed by a strong broad absorption peak around 3417–3421/cm. The peak located at 2931–2888/cm confirms the presence of C-H stretching group^17,59,60^. The absorption at 1730/cm corresponds to the stretch vibration of the carbonyl groups^53^. The recognized peak at 1603/cm is attributed to the carboxylate group, which characterizes the carbohydrate rings. Intense peaks at 1256 and 1024/cm are the main characteristic ones for polysaccharides. The extensive absorption bands at 1024/cm in the range of 1200–1000/cm in the FTIR spectrums is assigned to the C–O–C stretching vibration^59^. The weak absorption bands at 922/cm and 778/cm are attributed to glycosidic bonding of polysaccharides and skeleton bending of the pyranose ring, respectively^61^. The d-glucopyranoside, methyl 2,3,4,6-tetra-O-methyl—was the most profuse component (GC-Mass results) indicating that the backbone of the Pantoan was mainly composed of a (1–6) glycosidic linkage, in the previously published study about Pantoan derivatives^17^. Supplementary Fig. 2b depicts the XRD pattern of the biosynthetic Pantoan product. Although the product obtained in this study showed similar broad diffraction peaks, Pantoan produced with sucrose as substrate^17^ indicating their similar structure of the amorphous and small crystalline structures. In the food and medical industries, amorphous biopolymers may be more beneficial than value-added products such as edible films, coating agents, and drug carriers. The purity of the Pantoan was investigated after removing the proteins and nucleic acids which shed from the bacteria during fermentation. After purification through dialysis, the UV–vis absorption spectra method did not identify any proteins and nucleic acids (no distinctive absorption at 280 and 260 nm) (Supplementary Fig. 2c)^17^. This result indicates the absence of both nucleic acid and protein in the Pantoan EPS. The ^1^H NMR spectroscopy of the Pantoan from *Pantoea* sp. BCCS 001 GH is shown in (Supplementary Fig. 2d). The spectrum is in agreement with the carbohydrate composition of Pantoan, with two major chemical shifts in the anomeric region (4.5–5.5) were found (4.84 and 4.98) in ^1^H NMR^17,62^. The peak at δ 4.48 ppm and δ 4.98 ppm correspond to glucopyranosyl reducing end. Furthermore, both peak (4.84 and 4.98 ppm) are related to proton region of glucose and galactose monomers, respectively^17^. Moreover, the intensity of a doublet signal at 1.08 and 1.09 ppm is characteristic of typical substituent CH_3_-groups^63^. The thermal behavior of fermented product of *Pantoea* sp. BCCS 001 GH is crucially effective in its commercial applications. A weight loss of around 63.7% between 85 and 488 °C takes place in two stages:^64^ the initial weight loss of Pantoan was appeared between 85 and 170 °C due to loss of water, and (2) fast decomposition of Pantoan (16.6%) was observed between 170 and 488 °C, owing to Pantoan degradation (Supplementary Fig. 2e). According to the TGA curves, the maximum degradation temperature (Td) of Pantoan was 302 °C, similarly to 318 °C shown in a previous study^2^. The degradation temperature of the purified Pantoan in the present work that was produced with molasses as a substrate was slightly higher than other EPS such as ZW3 EPS, YW11 EPS, and succinoglycan 299.62, 287.7, and 261.04 °C, respectively^65–68^. These results indicate that Pantoan can maintain its structure during the food and pharmaceutical industrial thermo-processes, wherein temperatures seldom exceed 150 °C^69^. The DSC analysis of Pantoan showed both endothermic and exothermic peaks with heat flow from − 50 to 400 °C (Supplementary Fig. 2f). The first peak in the range of − 50 to 200 °C has appeared, contained an endothermic peak 146 °C of Pantoan, that corresponding to a melting point was due to the loss of absorbed and bound water. In general, the high value of melting point suggests that the Pantoan possessed a stronger ability to retain water, which was approved in the previous study^2^. The sample was thermally stable up to a relatively high 250 °C. However, the degradation temperature obtained in this work was 300 °C. Hence, given these results, the entirety of the thermal behavior data, we can conclude that introduction of produced Pantoan with molasses as substrate did not affect the ordering of polymer molecular chains compare to Pantoan produce with sucrose as substrate^2,17^. These results corroborate the thermostable nature of Pantoan up to 250 °C and its suitability to use in food and pharmaceutical industries.

**Figure 5:**
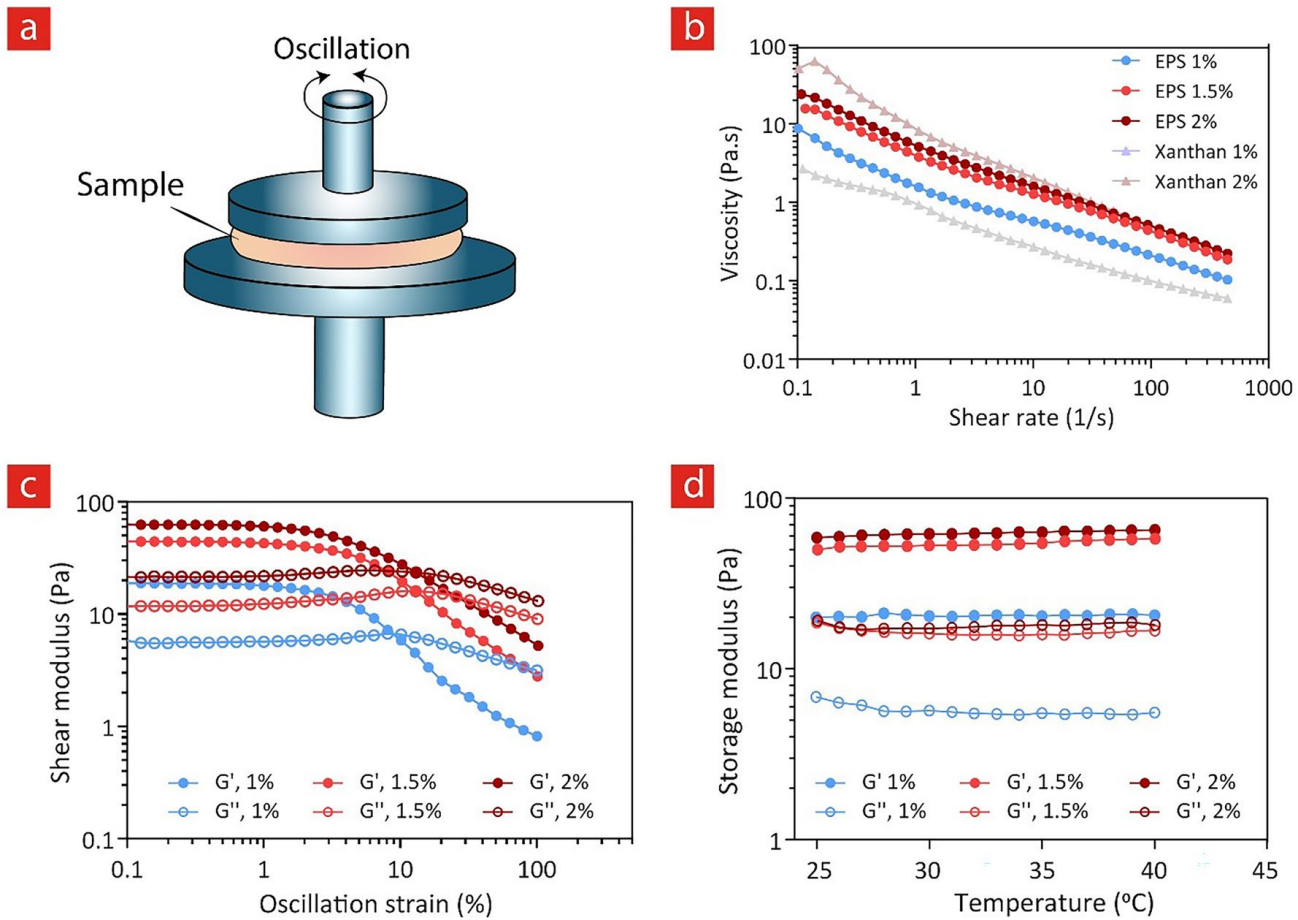
The rheological properties of Pantoan biopolymer solutions using GraphPad Prism 9 (GraphPad Software, San Diego, CA). (**a**) A schematic showing the parallel-plate geometries used to investigate rheological properties under oscillatory shear flow (**b**) Flow viscosity curves of Pantoan solutions produced by *Pantoea* sp. BCCS 001 GH at different concentrations the commercial Xanthan for comparicon (**c**) Amplitude sweeps and linear viscoelastic region curves of Pantoan with concentration series of 1, 1.5 and 2%. Storage moduli (G′) and loss moduli (G″) of amplitude sweep tests are plotted as a function of shear strain (**d**) Temperature sweep curves showing the storage and loss modulus of Pantoan solutions with different concentrations, between 25 and 40 °C.

The rheological behavior of polymeric solutions, when subjected to shear stress, is an important factor that defines what potential applications can be envisioned for newly introduced materials, such as EPSs. Specifically, there is an ever-increasing interest in these properties with the emergence of fabrication processes such as 3D printing in various industrial fields, where viscosity and shear-thinning behavior of the compound is of great importance. Therefore, a better understanding of the rheological behavior of the EPSs and the factors that affect this behavior opens up new avenues for the application of this natural biopolymer in the food industry and biomedical products^70^. Figure 5 demonstrates the shear-thinning characteristic of Pantoan solutions at different concentrations. The intertwined Pantoan molecules exhibit high resistance to flow at lower shear rates which corresponds to higher viscosity. However, upon increasing the shear rate, decreased viscosity is observed for all samples which stems from the alignment of macromolecules along the flow streamline^71^. As shown in this graph, the viscosity of 1% Pantoan at 0.01/s is around four times higher than that of 1% Xanthan, which suggests for less required material to obtain pseudo-plasticity at low concentrations. The viscosity of the 1% Pantoan solution remains higher than that of 1% Xanthan at all ranges of shear rate and the viscosity of Pantoan solutions was found to increase with increasing the concentrations. Altogether, these results suggest the Pantoan solution as a typical non-Newtonian solution. Shear-thinning solutions can induce a better mouthfeel due to their high pseudo-plasticity and can be easily pumped and transferred ^72^, therefore the produced Pantoan is suitable for food and pharmaceutical applications. Furthermore, to determine the linear viscoelastic (LVE) range of the polymer solutions, amplitude sweep test was conducted (Fig. 5c). Within the LVE strain range, the material can be deformed without any destructive effect in the structure, and this fundamental characteristic is an important parameter for any subsequent oscillatory measurements. The storage modulus (G′) showed strain-independent behavior and the loss modulus (G″) demonstrates a plateau for up to 5% oscillation strain in all concentrations. Moreover, it was observed from the temperature sweep curves that both storage and loss modulus of the polymer solutions remain constant within the temperature range of 25–40 °C. These results also indicate that the produced Pantoan can potentially be used as a bio-thickener and rheology modifiers and may find vast applications in the food, cosmetics, and pharmaceutical industries. Combining the results of the rheological investigation and thermal analysis shows that Pantoan is physically stable and can maintain its rheological properties even at high-temperature food and pharmaceutical processes.

## Conclusions

Production of microbial EPSs from an alternative and cheap carbon source is the recent interest of biopolymer research. Pantoan is an important extracellular bioactive molecule with both potential biological and antioxidant activities shown in our previous research. The present study is an extensive investigation on the optimization yield, growth kinetic, and characterization of Pantoan from *Pantoea* sp. BCCS 001 GH using SBM as a carbon source. The evolution of four significant effective factors for Pantoan production, in other words, SBM, peptone, Na_2_HPO_4_, and Triton X-100 were investigated. The maximum yield of Pantoan (9.9 ± 0.5 g/L) was obtained after 48 h which make it suitable bacteria to produce value-added biopolymer in industry. Fundamental unstructured kinetic model equations exactly fitting to the experiment’s data (actual data) were performed to learn the dynamics of cell growth, SBM consumption, and Pantoan production by the *Pantoea* sp. BCCS 001 GH. Moreover, it was demonstrated that the characterization of the Pantoan fraction was mainly composed of galactose and glucose. In summary, the presence of unique sugars in the Pantoan structure, with its high production capacity, makes this biopolymer interesting for further innovative development, particularly in health food and biomedical fields.

## Supplementary Information

Supplementary Information.

## Abbreviations

SBM: Sugar beet molasses
RSM: Response surface methodology
CCD: Central composite design
Td: Degradation temperature
EPS: Exopolysaccharides
DNS: Dinitrosalicylic acid
AAS: Atomic absorption spectrophotometer
x: Cell mass concentration
p: Product concentration
s: Sugar concentration of substrate
UV–Vis: Ultraviolet–Visible
XRD: X-ray diffraction
TGA: Thermogravimetric analysis
DSC: Dynamic variance scanning calorimetry
ANOVA: Analysis of variance
