# Supplementary material for: Biosynthesis of exopolysaccharide from waste molasses using Pantoea sp. BCCS 001 GH: a kinetic and optimization study
Source: Sci Rep. 2022 Jun 16;12:10128. doi: 10.1038/s41598-022-14417-1 (PMC9203581; doi:10.1038/s41598-022-14417-1)
Supplement: Supplementary file 1 — Supplementary Information. [file 41598_2022_14417_MOESM1_ESM.docx]

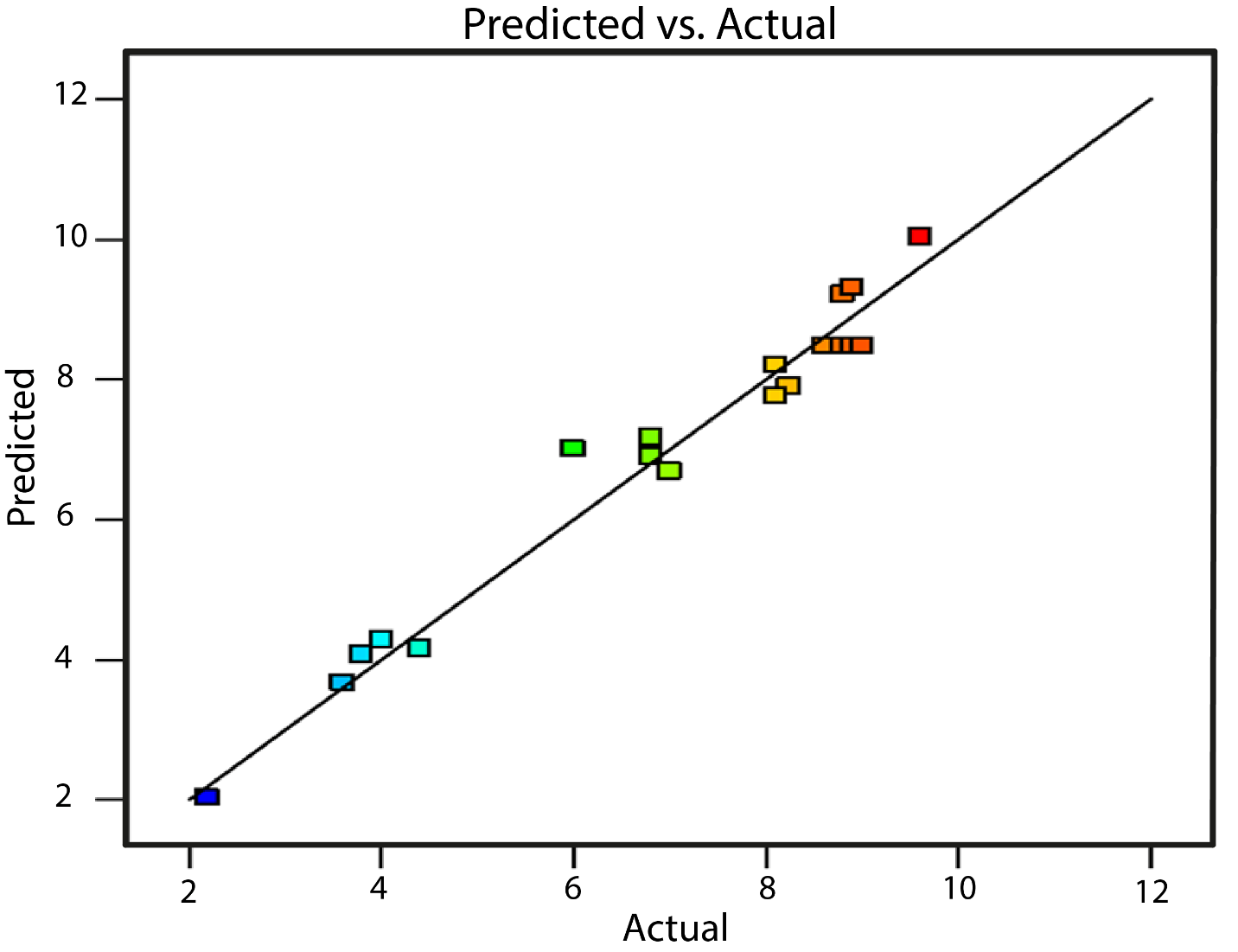


**Supplementary Figure 1**. Parity plot: showing the relation between actual and predicted values for Pantoan elaboration


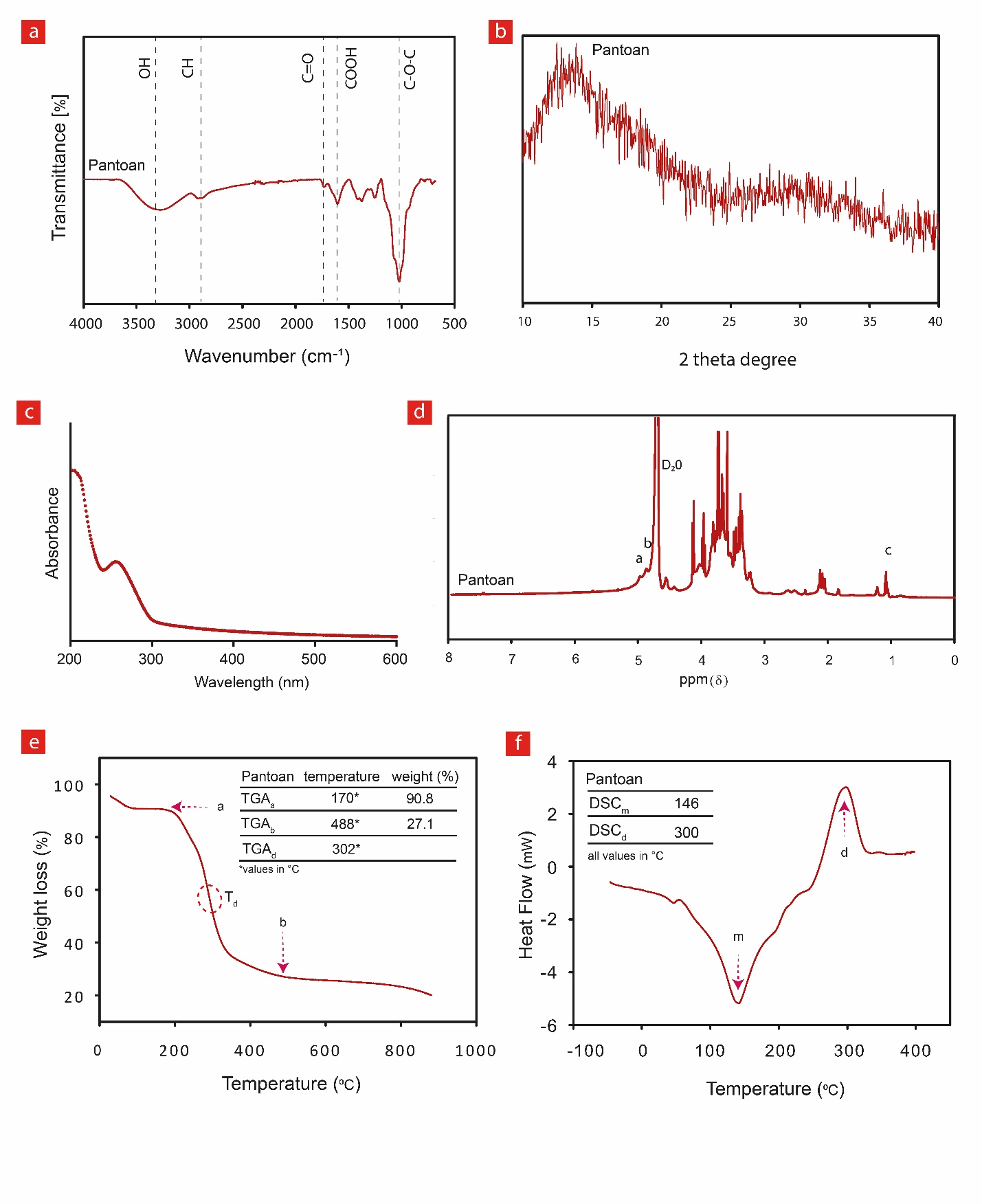


**Supplementary Figure 2**. Characterization of Pantoan produced with molasses as substrate. (**a**) FTIR, (**b**) XRD, (**c**) Ultraviolet–visible (UV–vis) spectroscopy analysis of the Pantoan, (**d**) H^1^ NMR, (**e**) TGA, and (**f**) DSC.

**Supplementary Table 1**. Comparison of yields between optimized and non-optimized media for Pantoan production

| **Batch number** | **A: SBM (g/L)** | **B: Peptone (g/L)** | | **C: Na_2_HPO_4_ (g/L)** | **D: Triton X-100 (g/L)** | **Pantoan (g/L)** |
| --- | --- | --- | --- | --- | --- | --- |
| Batch 1 (non-optimized) | 10 | | 3 | 3 | 0.2 | 5 ± 0.25 |
| Batch 2 (non-optimized) | 15 | | 3 | 3 | 0.2 | 5.7 ± 0.3 |
| Batch 3 (non-optimized) | 20 | | 3 | 3 | 0.2 | 5.6 ± 0.4 |
| Batch 4 (non-optimized) | 30 | | 3 | 3 | 0.2 | 7.3 ± 0.25 |
| Batch 5 (non-optimized) | 40 | | 3 | 3 | 0.2 | 8.0 ± 0.2 |
| Batch 6 (optimized) | 31.5 | | 2.73 | 3 | 0.32 | 9.9 ± 0.5 |
